# Syphilitic Panuveitis Presenting as Bilateral Granulomatous Uveitis in an Immunocompetent Patient: A Case Report

**DOI:** 10.7759/cureus.106619

**Published:** 2026-04-07

**Authors:** Battuya Ganbold, Bayasgalan Purevdorj, Dariimaa Ganbat

**Affiliations:** 1 Department of Ophthalmology, Bolor Melmii Hospital, Center of International Cyber Education, Graduate School of Medical Science, Mongolian National University of Medical Sciences, Ulaanbaatar, MNG; 2 Department of Ophthalmology, Mongolian National University of Medical Sciences, Ulaanbaatar, MNG; 3 Center of International Cyber Education, Graduate School of Medical Science, Mongolian National University of Medical Sciences, Ulaanbaatar, MNG

**Keywords:** case report, granulomatous uveitis, ocular syphilis, panuveitis, syphilitic uveitis, treponema pallidum

## Abstract

Ocular syphilis is a re-emerging, sight-threatening manifestation of *Treponema pallidum* infection that can mimic a wide range of inflammatory eye diseases. We report the case of a 31-year-old immunocompetent woman who presented with a two-month history of progressive bilateral blurred vision, ocular pain, redness, photophobia, and floaters. Ophthalmic examination revealed bilateral granulomatous panuveitis with mutton-fat keratic precipitates, anterior chamber inflammation, vitritis, optic disc hyperemia, and multifocal chorioretinal lesions. Dermatologic examination demonstrated non-pruritic maculopapular eruptions on both palms and the right sole, raising suspicion for secondary syphilis. Serologic testing showed a rapid plasma reagin (RPR) titer of 1:320 and a positive *Treponema pallidum* hemagglutination assay (TPHA), while human immunodeficiency virus (HIV) and tuberculosis screening were negative. Optical coherence tomography (OCT) demonstrated preserved foveal architecture without cystoid macular edema. Due to the unavailability of standard intravenous therapy in the local clinical setting, the patient was treated with a locally adapted, non-standard regimen of intramuscular benzathine penicillin G 2.4 million units at seven-day intervals for three doses, along with topical and systemic corticosteroids. By day 28, best-corrected visual acuity improved to 20/20 in both eyes, with marked resolution of inflammation. At three months, the RPR titer declined fourfold, indicating an adequate serologic response. This case highlights the importance of considering syphilis in the differential diagnosis of bilateral granulomatous panuveitis, particularly when accompanied by a palmoplantar rash. It underscores the value of prompt serologic testing and timely treatment to preserve vision, while also acknowledging the challenges of adhering to standard treatment protocols in resource-limited settings.

## Introduction

Ocular syphilis is a potentially vision-threatening manifestation of *Treponema pallidum* infection that can occur at any stage of syphilis and involve virtually any ocular structure [1-5]. Because its clinical manifestations are protean, ocular syphilis may present as anterior, intermediate, posterior, or panuveitis. It can also mimic numerous infectious and non-infectious causes of intraocular inflammation, making it an important diagnostic masquerader in patients with unexplained uveitis [1-6]. Over the past two decades, a resurgence of syphilis has been documented globally, with increasing reports of ocular involvement across diverse populations and clinical settings [2,3,7,8]. Early recognition is essential because delayed diagnosis and treatment may result in irreversible visual impairment, including retinal or optic nerve injury, whereas timely therapy is often associated with favorable visual outcomes [4-6,9,10].

Current CDC sexually transmitted infections treatment guidelines recommend urgent ophthalmologic evaluation when ocular syphilis is suspected and management using neurosyphilis treatment recommendations, even when cerebrospinal fluid findings are normal [7]. In this context, our case is clinically relevant because it highlights bilateral granulomatous panuveitis as the presenting manifestation of syphilitic uveitis in an immunocompetent young woman with secondary syphilis, emphasizing the diagnostic value of careful systemic examination and prompt serologic testing [1-3,5].

## Case presentation

A 31-year-old previously healthy woman presented with a two-month history of progressive bilateral visual impairment, ocular pain, and redness. Her symptoms began in the left eye and involved the right eye two weeks later. She also reported photophobia and floaters. She denied ocular trauma, recent systemic infection, or known tuberculosis exposure. Her past medical history was unremarkable, with no history of uveitis or autoimmune disease. She was not taking any regular medications and had no known drug allergies. Although she denied a previous history of sexually transmitted infection, a detailed sexual history was not obtained at the initial presentation.

**Figure 1:**
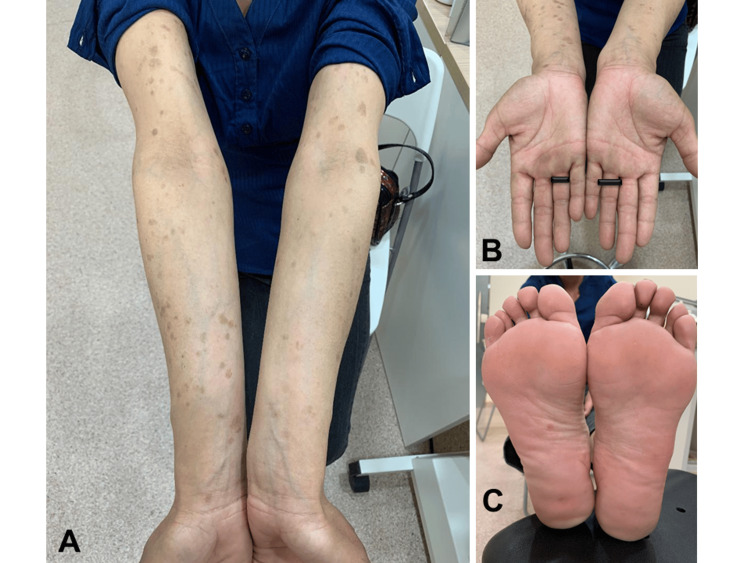
Palmoplantar and forearm rash in secondary syphilis. Clinical photographs showing (A) brownish maculopapular lesions on the forearms, (B) bilateral palmar lesions, and (C) plantar lesions on the right sole. These non-pruritic palmoplantar and forearm eruptions supported the diagnosis of secondary syphilis in this patient with ocular involvement.

On examination, best-corrected visual acuity (BCVA) was 20/30 in the right eye and 20/40 in the left eye. Slit-lamp examination revealed bilateral granulomatous anterior uveitis with multiple mutton-fat keratic precipitates and 2+ cells in the right anterior chamber and 1+ cells in the left. Posterior synechiae were present in the left eye. Intraocular pressure was normal in both eyes. Dilated fundus examination revealed bilateral panuveitis with moderate vitritis, optic disc hyperemia with blurred margins, mild retinal perivascular sheathing, and multiple pale yellow chorioretinal lesions involving the macula and peripheral retina. General physical examination revealed well-demarcated, brownish, non-pruritic maculopapular rashes on both palms and the plantar surface of the right foot, which strongly suggested secondary syphilis (Figure 1).

**Figure 2:**
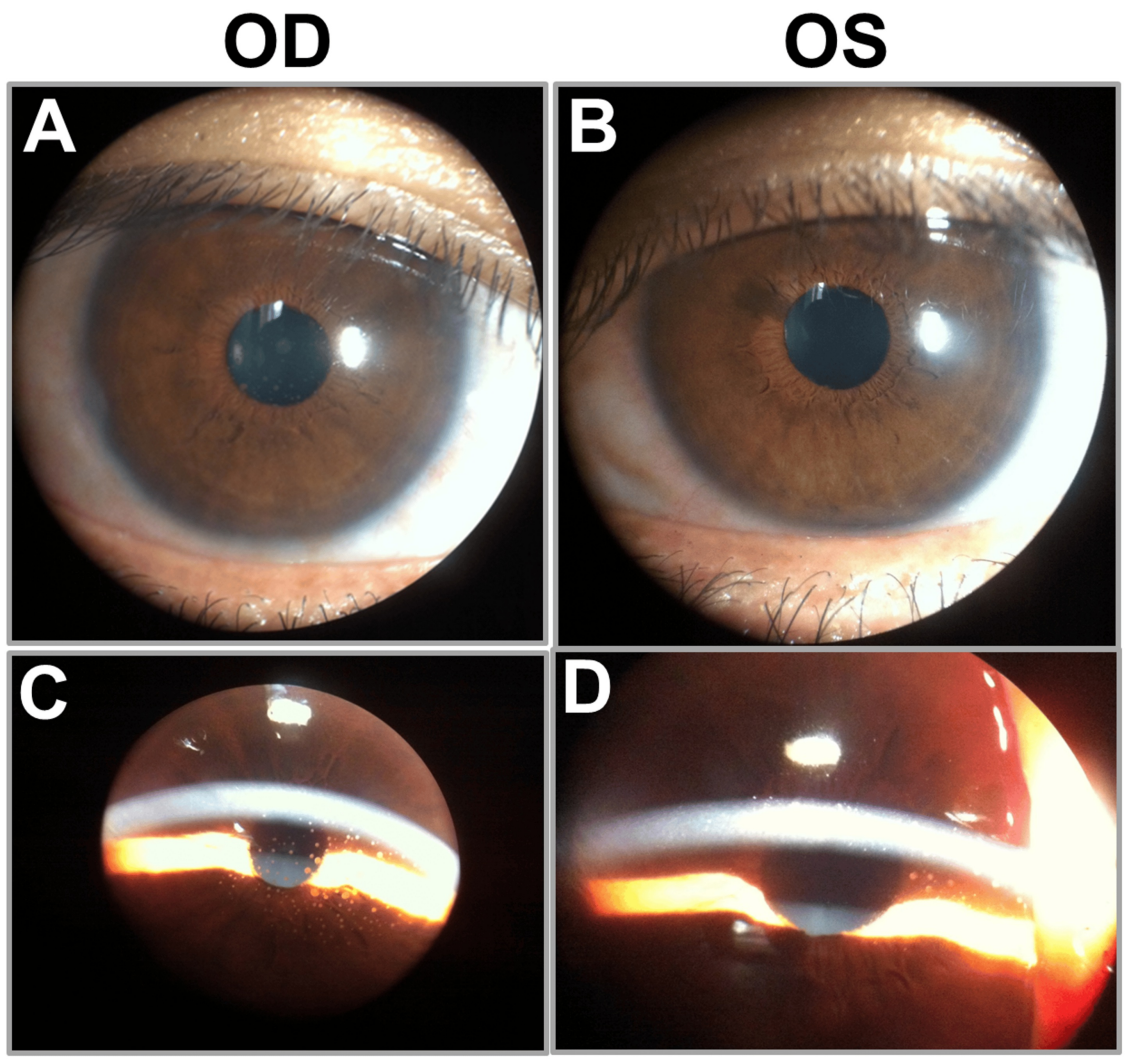
Anterior segment findings in bilateral syphilitic panuveitis. Anterior segment photographs of both eyes showing granulomatous anterior uveitis. (A and B) Right eye with mutton-fat keratic precipitates and anterior chamber inflammation. (C and D) Left eye with mutton-fat keratic precipitates, anterior chamber inflammation, and posterior synechiae. Arrowheads indicate the principal inflammatory findings.

**Figure 3:**
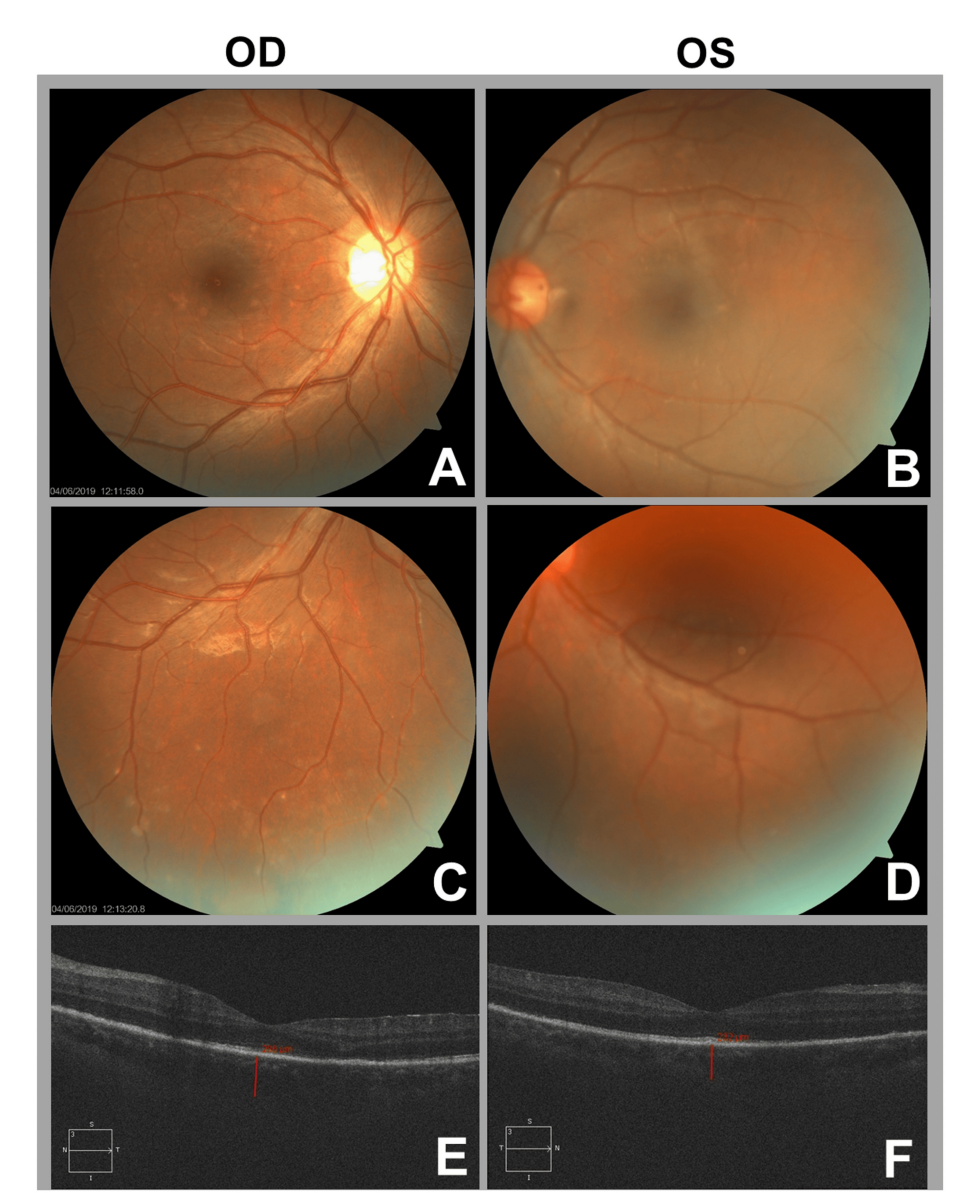
Ocular findings in bilateral syphilitic panuveitis. Fundus photographs (A-D) demonstrate optic disc hyperemia, vitritis, mild retinal perivascular sheathing, and multifocal pale yellow chorioretinal lesions in both eyes. Macular OCT (E and F) shows preserved foveal contour without cystoid macular edema or subretinal fluid. OCT: optical coherence tomography.

A comprehensive laboratory evaluation was performed. Syphilis serology was strongly positive, with a rapid plasma reagin (RPR) titer of 1:320 and a positive *Treponema pallidum* hemagglutination assay (TPHA) result of 292.00 COI, confirming active syphilitic infection. Human immunodeficiency virus (HIV) testing was negative. Tuberculosis screening, including an interferon-gamma release assay and chest radiography, was negative. Additional investigations, including complete blood count, erythrocyte sedimentation rate, antinuclear antibody, angiotensin-converting enzyme level, and Toxoplasma serology, were unremarkable. OCT showed preserved foveal architecture in both eyes without cystoid macular edema or subretinal fluid. Fundus photography documented the bilateral optic disc hyperemia, vitritis, and chorioretinal lesions. Based on the ocular findings, characteristic palmoplantar rash, and confirmatory serology, a diagnosis of bilateral syphilitic panuveitis secondary to secondary syphilis was established (Figures 2-3).

**Figure 4:**
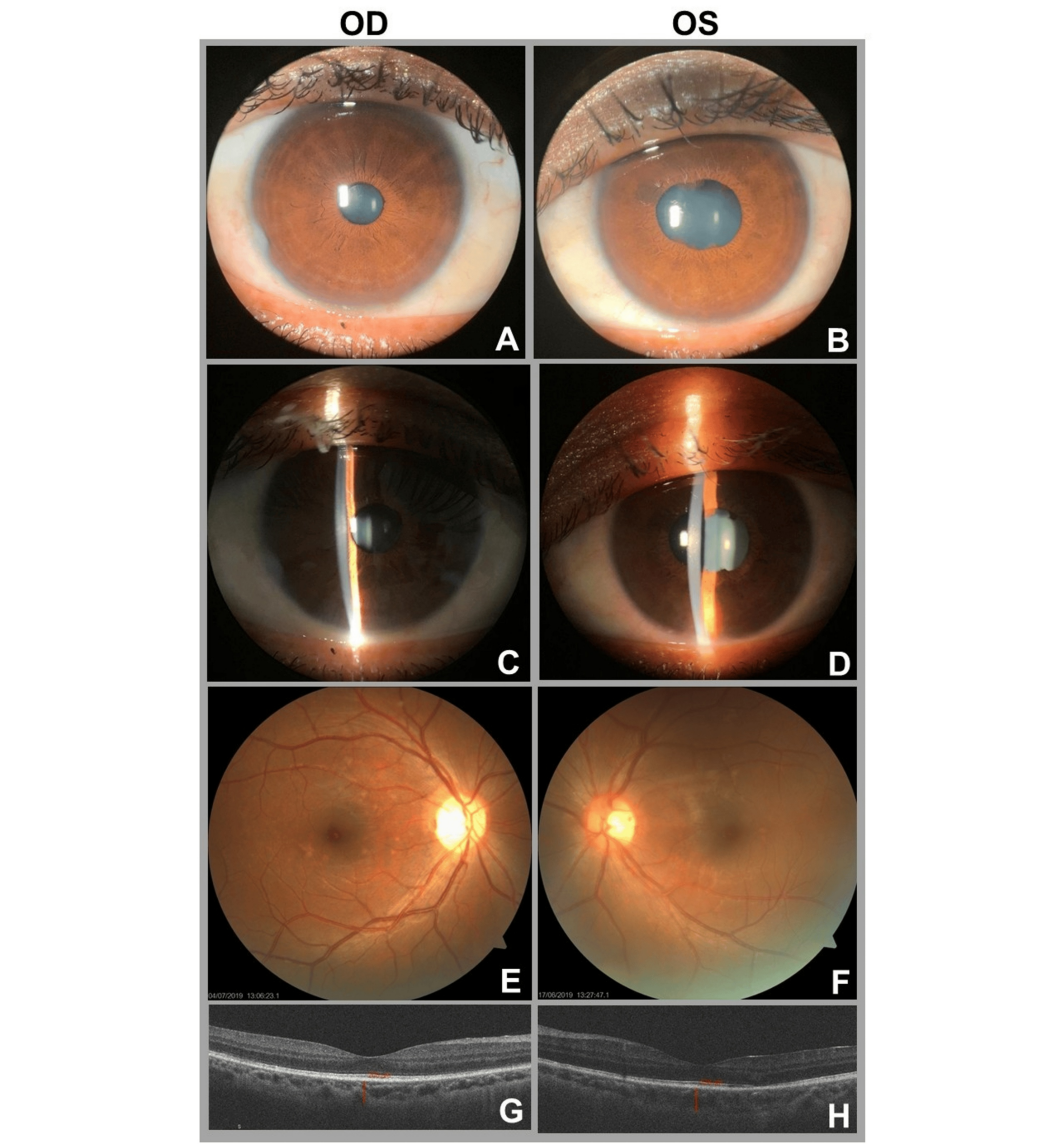
One-month follow-up ocular findings after initiation of treatment for bilateral syphilitic panuveitis. Anterior segment, fundus, and optical coherence tomography (OCT) images obtained one month after treatment initiation. (A, C, E, and G) Right eye (OD). (B, D, F, and H) Left eye (OS). (A-D) Anterior segment photographs show marked improvement in granulomatous anterior uveitis, with resolution of keratic precipitates and reduction of anterior chamber inflammation; residual posterior synechiae remain visible in the left eye. (E and F) Fundus photographs demonstrate interval improvement in vitreous haze and posterior segment inflammation, with resolution of optic disc hyperemia and decreased visibility of prior chorioretinal inflammatory lesions. (G and H) Macular OCT images show preserved foveal contour without cystoid macular edema or subretinal fluid. OCT: optical coherence tomography

Topical treatment was initiated with prednisolone acetate 1% every two hours and homatropine (Homide) twice daily in both eyes. After serologic confirmation of syphilis, systemic therapy was started with benzathine penicillin G 2.4 million units intramuscularly at seven-day intervals for three doses, as standard intravenous therapy for neurosyphilis was not available in the local clinical setting. Additional treatment included doxycycline 100 mg twice daily for 14 days, ciprofloxacin 400 mg once daily for 14 days, and clotrimazole vaginal suppositories. Oral prednisolone (40 mg/day) was introduced three days after initiation of antimicrobial therapy and was gradually tapered. The patient was counseled regarding the infectious nature of syphilis, the importance of partner notification, adherence to treatment, and follow-up. At the one-month follow-up after treatment initiation, multimodal ocular examination demonstrated marked interval improvement in bilateral syphilitic panuveitis, with resolution of keratic precipitates, reduction of anterior chamber and vitreous inflammation, improvement in posterior segment findings, and preserved foveal architecture on OCT without cystoid macular edema or subretinal fluid (Figure 4).

The patient's clinical response was excellent. By week 1, she reported improved vision and decreased pain. At four weeks, BCVA had improved to 20/20 in both eyes, with only trace residual inflammation. OCT continued to demonstrate preserved macular architecture. At three-month follow-up, visual acuity remained 20/20, inflammation had completely resolved, and a repeat RPR titer showed a fourfold decline to 1:80, consistent with an appropriate serologic response to treatment. The clinical course is summarized in Table 1, which provides a structured timeline of the major symptoms, diagnostic findings, treatment milestones, and follow-up outcomes.

**Table 1:** Timeline of the clinical course. Visual acuity is reported in decimal notation. BCVA: best-corrected visual acuity; OD: right eye; OS: left eye; OCT: optical coherence tomography; IOP: intraocular pressure; RPR: rapid plasma reagin; TPHA: *Treponema pallidum* hemagglutination assay; HIV: human immunodeficiency virus; TB: tuberculosis.

Time point	Clinical event
Eight weeks before presentation	Onset of left-eye redness, pain, and blurred vision.
Six weeks before presentation	Progressive worsening of left-eye symptoms with the development of floaters. A brownish rash appeared on both forearms/palms and a faded rash on the right sole.
Four weeks before presentation	Onset of right-eye redness, pain, and blurred vision, approximately two weeks after the left eye became symptomatic.
Two weeks before presentation	Progressive bilateral visual deterioration, prompting medical consultation.
Day 0 (initial presentation)	A 31-year-old immunocompetent woman underwent comprehensive ophthalmic examination. Visual acuity was 0.5 in both eyes; BCVA was 0.7 in the right eye and 0.5 in the left eye. Slit-lamp examination showed mutton-fat keratic precipitates in both eyes, anterior chamber cells (+2 OD, +1 OS), and posterior synechiae in the left eye. Intraocular pressure was 8 mmHg OD and 9 mmHg OS. OCT showed preserved foveal architecture, with central foveal thickness of 239 µm OD and 259 µm OS. The initial diagnosis was bilateral granulomatous panuveitis. Blood tests were ordered, including RPR, TPHA, HIV, Quantiferon-TB Gold, complete blood count, erythrocyte sedimentation rate, fasting blood glucose, and chest radiography.
Day 3	Serologic testing confirmed syphilis with RPR 1:320 and TPHA 292.00 COI (positive); HIV and tuberculosis screening were negative. The diagnosis was revised to syphilitic panuveitis associated with secondary syphilis. Topical treatment included prednisolone acetate 1% every two hours and homatropine (Homide) twice daily. Systemic treatment consisted of benzathine penicillin G 2.4 million units intramuscularly at seven-day intervals for three doses, doxycycline 100 mg twice daily for 14 days, ciprofloxacin 400 mg once daily for 14 days, and clotrimazole vaginal suppositories.
Day 6	Oral prednisolone 40 mg daily and omeprazole 20 mg daily were started three days after initiation of antibiotic therapy.
Day 7	First follow-up: subjective improvement in vision and ocular pain, with reduction in anterior chamber inflammation.
Day 14	Second follow-up: continued improvement, with reduction in vitritis; visual acuity improved to 0.7 bilaterally.
Day 21	Third follow-up: significant resolution of keratic precipitates and vitreous cells.
Day 28	Fourth follow-up: visual acuity improved to 1.0 (20/20) in both eyes, with marked improvement in anterior and posterior segment inflammation; OCT showed preserved foveal architecture.
Three months after treatment	Long-term follow-up showed maintained visual acuity of 1.0 bilaterally, complete resolution of inflammation, and no recurrence.

The chronological sequence of bilateral ocular symptoms, granulomatous intraocular inflammation on examination, supportive OCT findings, systemic cutaneous manifestations, and confirmatory serologic testing allowed timely diagnosis of syphilitic panuveitis associated with secondary syphilis. Serial follow-up examinations demonstrated progressive reduction of intraocular inflammation with recovery of visual acuity to 1.0 in both eyes and no recurrence at three months.

## Discussion

Ocular syphilis remains a re-emerging cause of potentially sight-threatening uveitis and continues to pose a diagnostic challenge because it can mimic a broad spectrum of inflammatory ocular disorders. Case series from Malaysia and India have shown that panuveitis and bilateral involvement are common manifestations, while broader reviews emphasize that syphilitic uveitis may affect nearly any ocular structure [2,3,5]. In this context, our patient's bilateral granulomatous panuveitis with vitritis, papillitis, and multifocal chorioretinal lesions is well within the recognized clinical spectrum of ocular syphilis [2,3,5].

A central diagnostic lesson in this case is the importance of careful systemic examination. The palmoplantar maculopapular rash was the critical clue that narrowed the differential diagnosis. The differential diagnosis of bilateral granulomatous panuveitis in this patient included TB, sarcoidosis, toxoplasmosis, and other infectious or non-infectious inflammatory uveitides. However, TB screening, including interferon-gamma release assay and chest radiography, was negative; serum angiotensin-converting enzyme level was unremarkable; toxoplasma serology was negative; and the characteristic palmoplantar rash, together with strongly positive non-treponemal and treponemal serology, strongly supported ocular syphilis as the most likely diagnosis. This point is clinically important because missed opportunities to recognize syphilis before the onset of sight-threatening uveitis have been reported [5,10]. Once suspected, ocular syphilis should be confirmed serologically using both non-treponemal and treponemal tests. In our patient, the high RPR titer with a positive TPHA established an active infection, and the subsequent fourfold decline in RPR supported an appropriate serologic response to treatment [7,11].

The favorable visual outcome in this patient was likely related to prompt diagnosis and treatment before irreversible macular or optic nerve damage developed. OCT demonstrating preserved foveal architecture was a reassuring prognostic feature. This outcome aligns with previous reports showing that early treatment of ocular syphilis can result in substantial visual recovery [4,6,9,10].

However, the treatment course in this case should be interpreted with caution. Current CDC guidance recommends managing ocular syphilis according to neurosyphilis treatment protocols, preferably with intravenous aqueous crystalline penicillin G for 10-14 days, with intramuscular procaine penicillin G plus oral probenecid as an alternative regimen in selected cases [7]. In our patient, the regimen used was a locally adapted, non-standard approach because standard intravenous therapy was unavailable in the local clinical setting. Although the patient showed favorable clinical and serologic improvement, this outcome should not be interpreted as evidence that the regimen used here is equivalent to recommended guideline-based therapy. Adjunctive topical and systemic corticosteroids were introduced only after antimicrobial therapy had been initiated to control inflammation while monitoring the clinical response [11-13].

This case should be interpreted in light of several limitations. First, cerebrospinal fluid analysis was not performed; therefore, concurrent neurosyphilis could not be formally assessed. Although ocular syphilis is treated according to neurosyphilis recommendations regardless of CSF findings, the absence of CSF examination limits complete neurologic correlation in this case. Second, the duration of follow-up was relatively short, which limits assessment of long-term visual and anatomic outcomes as well as recurrence risk. Nevertheless, the combination of bilateral granulomatous panuveitis, characteristic systemic cutaneous findings, positive serologic testing, exclusion of HIV and TB, and favorable response to treatment strongly supported the diagnosis and clinical interpretation.

## Conclusions

This case underscores the importance of considering syphilitic panuveitis in the differential diagnosis of bilateral uveitis, even in immunocompetent patients. Careful systemic examination, recognition of associated mucocutaneous findings, and prompt serologic testing were essential for establishing the diagnosis. Although this patient showed favorable clinical and serologic improvement after treatment, the result should be interpreted cautiously because the regimen used was non-standard and was selected because of local treatment limitations. Furthermore, the absence of CSF analysis and the relatively short duration of follow-up limit generalization from this single case. Greater clinical awareness of ocular syphilis may help reduce diagnostic delay and preserve vision.
